# Tyrosine Sulfation of the Amino Terminus of PSGL-1 Is Critical for Enterovirus 71 Infection

**DOI:** 10.1371/journal.ppat.1001174

**Published:** 2010-11-04

**Authors:** Yorihiro Nishimura, Takaji Wakita, Hiroyuki Shimizu

**Affiliations:** Department of Virology II, National Institute of Infectious Diseases, Musashimurayama-shi, Tokyo, Japan; Harvard Medical School, United States of America

## Abstract

Enterovirus 71 (EV71) is one of the major causative agents of hand, foot, and mouth disease, a common febrile disease in children; however, EV71 has been also associated with various neurological diseases including fatal cases in large EV71 outbreaks particularly in the Asia Pacific region. Recently we identified human P-selectin glycoprotein ligand-1 (PSGL-1) as a cellular receptor for entry and replication of EV71 in leukocytes. PSGL-1 is a sialomucin expressed on the surface of leukocytes, serves as a high affinity counterreceptor for selectins, and mediates leukocyte rolling on the endothelium. The PSGL-1–P-selectin interaction requires sulfation of at least one of three clustered tyrosines and an adjacent *O*-glycan expressing sialyl Lewis x in an N-terminal region of PSGL-1. To elucidate the molecular basis of the PSGL-1–EV71 interaction, we generated a series of PSGL-1 mutants and identified the post-translational modifications that are critical for binding of PSGL-1 to EV71. We expressed the PSGL-1 mutants in 293T cells and the transfected cells were assayed for their abilities to bind to EV71 by flow cytometry. We found that *O*-glycosylation on T57, which is critical for PSGL-1–selectin interaction, is not necessary for PSGL-1 binding to EV71. On the other hand, site-directed mutagenesis at one or more potential tyrosine sulfation sites in the N-terminal region of PSGL-1 significantly impaired PSGL-1 binding to EV71. Furthermore, an inhibitor of sulfation, sodium chlorate, blocked the PSGL-1–EV71 interaction and inhibited PSGL-1-mediated viral replication of EV71 in Jurkat T cells in a dose-dependent manner. Thus, the results presented in this study reveal that tyrosine sulfation, but not *O*-glycosylation, in the N-terminal region of PSGL-1 may facilitate virus entry and replication of EV71 in leukocytes.

## Author Summary

Enterovirus 71 (EV71) is a major causative agent of hand, foot, and mouth disease and a diverse array of neurological diseases, including fatal encephalitis, in children. EV71 has increasingly caused large outbreaks of hand, foot, and mouth disease particularly in the Asia-Pacific region. Recently, we identified human P-selectin glycoprotein ligand-1 (PSGL-1) as a functional receptor for EV71. PSGL-1 on immune cells is a key molecule involved in early inflammatory events and the PSGL-1–selectin interaction is regulated by post-translational modifications of PSGL-1. Here, we found that a post-translational modification, tyrosine sulfation, at the N-terminal region of PSGL-1 is critical for its binding to EV71 and subsequent viral replication in lymphocytes. Important roles for tyrosine sulfation in protein-protein interactions have been widely accepted; however, involvement of tyrosine sulfation of the receptor in the virus-receptor interaction has been reported only for HIV-1. Therefore, this is the second and unique example of the involvement of tyrosine sulfation in specific virus-receptor interactions. Our results shed new light on biological roles for tyrosine-sulfated proteins in cell tropism and the pathogenesis of EV71.

## Introduction

Enterovirus 71 (EV71) is a small, nonenveloped, positive-stranded RNA virus that belongs to human enterovirus species A of the genus *Enterovirus* in the family *Picornaviridae*. EV71 is a major causative agent of hand, foot, and mouth disease (HFMD), a common febrile disease affecting mainly young children. HFMD is characterized by a skin rash on the palms and soles, and ulcers on the oral mucosa. HFMD due to EV71 and other enteroviruses is usually mild and self-limited; however, EV71 infection may also cause severe neurological diseases including polio-like paralysis and fatal brainstem encephalitis in young children and infants (reviewed in [1], [2]). Over the last decade, many EV71 outbreaks involving a number of fatal encephalitis cases have been reported throughout the world, especially in the Asia-Pacific region, including in Malaysia, Taiwan, Vietnam, and mainland China [2], [3], [4].

Using an expression cloning method by panning with a cDNA library from human Jurkat T cells, we recently identified human P-selectin glycoprotein ligand-1 (PSGL-1) as a functional cellular receptor for EV71 [5]. In addition, Yamayoshi et al. [6] identified scavenger receptor class B, member 2 (SCARB2) as another cellular receptor for EV71 by screening EV71-susceptible transformants after transfecting mouse L929 cells with genomic DNA from human RD rhabdomyosarcoma cells. SCARB2 is ubiquitously expressed on a variety of tissues and cells [7], whereas the tissue distribution of PSGL-1 is mainly limited to immune cells such as leukocytes and dendritic cells [8]. We have also demonstrated that some EV71 strains (PSGL-1–binding strain; EV71-PB) use PSGL-1 as the primary and functional receptor for infection of Jurkat T cells, but other EV71 strains (PSGL-1–non-binding strain; EV71-non-PB) do not, suggesting phenotypic differences in PSGL-1 usage among EV71 strains. Thus, the identification of two distinct cellular receptors for EV71, PSGL-1 and SCARB2, has provided important clues in the elucidation of the molecular basis of early virus-host interactions and pathogenesis of EV71. However, little is known about the biological significance of the two EV71 receptors.

PSGL-1 is a sialomucin membrane protein that is expressed as a homodimer comprised of two disulfide-linked subunits. Interaction of PSGL-1 with selectins and chemokines is a key event during early inflammation of immune cells [8], [9], [10], [11]. The N-terminal region of PSGL-1 is critical for PSGL-1 binding to P-, E- and L-selectins, and post-translational modifications such as *O*-glycosylation and tyrosine sulfation in the N-terminal region of PSGL-1 contribute the efficient binding to selectins [12], [13], [14], [15]. We have previously shown that the N-terminal region of human PSGL-1 (amino acids 42–61) containing a potential *O*-glycosylation residue (T57) and three potential tyrosine sulfation sites (Y46, Y48, and Y51) is directly responsible for PSGL-1 binding to EV71-PB [5]. Therefore, in the present study, we investigated the involvement of post-translational modifications of PSGL-1 in the binding to EV71-PB using a series of PSGL-1 mutants and an inhibitor of sulfation.

Tyrosine sulfation is an important late post-translational modification of secreted and membrane-bound proteins expressed in various mammalian cells and tissues and occurs in the trans-Golgi network [16], [17]. Tyrosine sulfated proteins have been described in many mammalian species, and important roles for tyrosine sulfation in protein-protein interactions have been widely accepted, particularly for various chemokine receptors and their ligands that mediate leukocyte migration during inflammation. Furthermore, it has been well established that tyrosine sulfation of the N-terminal region of the chemokine receptor, C-C chemokine receptor 5 (CCR5), plays critical roles in the function of CCR5 as a coreceptor for virus entry and replication of CCR5-tropic human immunodeficiency virus type 1 (HIV-1) variants [18].

Here we demonstrate that tyrosine sulfation of the N-terminal region of PSGL-1 facilitates PSGL-1–EV71 interaction and viral replication of EV71-PB in Jurkat T cells. To our knowledge, this is the second direct example of the involvement of tyrosine sulfation in specific virus-receptor interactions, a modification that mediates viral entry and replication in target cells.

## Results

### *O*-glycosylation at T57 of PSGL-1 is not necessary for EV71-1095 binding

**Figure 1:**
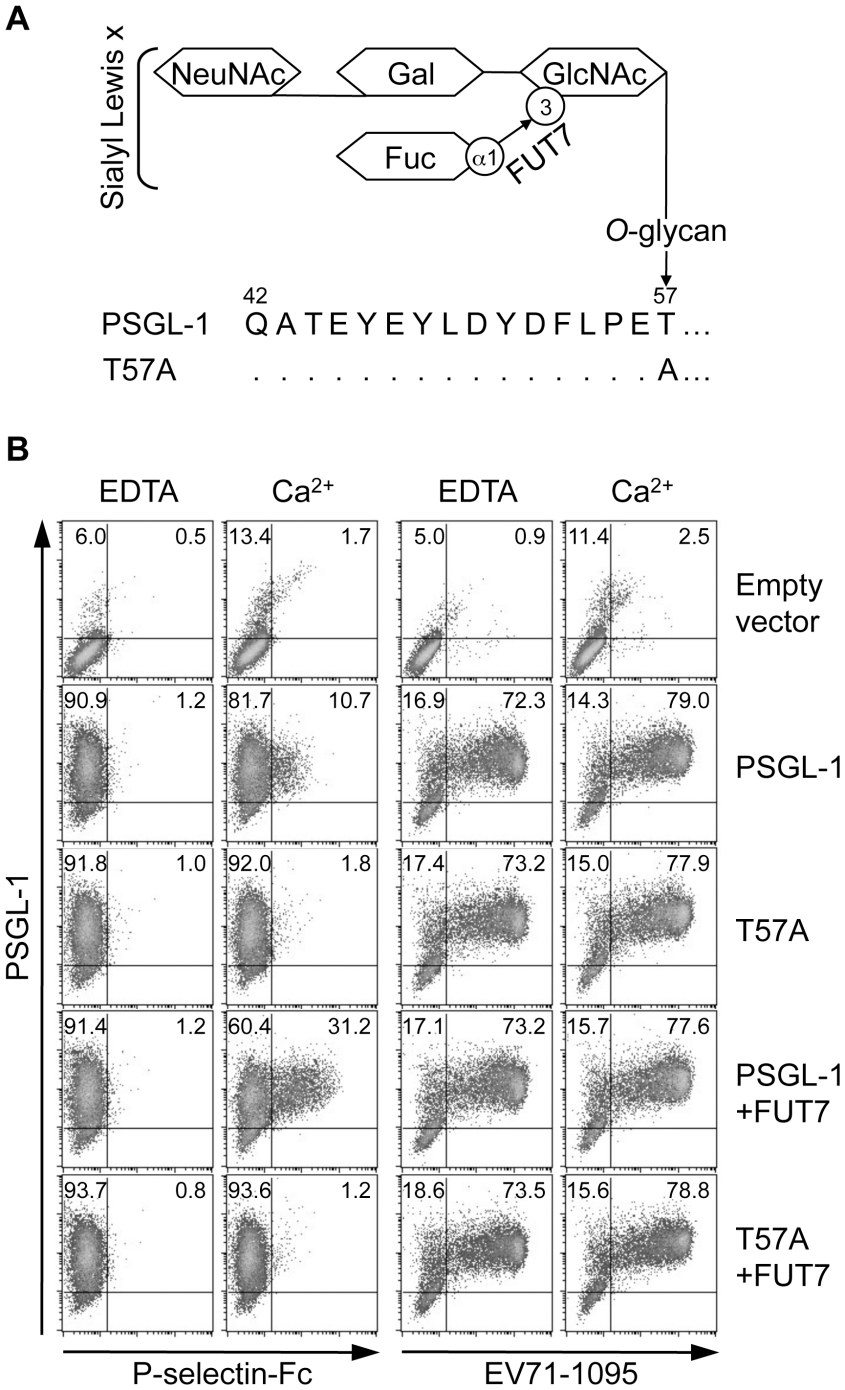
PSGL-1 *O*-glycosylation at T57 is not necessary for binding to EV71-1095. (A) Schematic structure of the *O*-glycosylation of PSGL-1 and the T57A mutant. FUT7 is involved in the synthesis of sialyl Lewis x. (B) 293T cells were transfected with the indicated expression plasmids. Transfectants were incubated with P-selectin-Fc or EV71-1095 in the presence (Ca^2+^) or absence (EDTA) of 2 mM CaCl_2_ followed by the P-selectin-Fc or EV71 binding assay using flow cytometry. The percentage of cells bound to P-selectin-Fc or EV71-1095 is indicated in the upper right quadrant. The data are representative of three independent experiments.

For binding to selectins, PSGL-1 requires post-translational modifications with sialyl Lewis x-containing *O*-glycans at T57. α1,3-fucosyltransferase (FUT7) is involved in the biosynthesis of sialyl Lewis x determinants (Fig. 1A) [19], [20]. Prevention of *O*-glycosylation by alanine substitution at T57 (T57A) eliminates binding of PSGL-1 to P-selectin without affecting tyrosine sulfation [12]. First, we generated and expressed a PSGL-1-T57A mutant (Fig. 1A) in 293T cells (293T/T57A) to examine the role of *O*-glycosylation on T57 for PSGL-1 binding to EV71-1095, a representative strain of EV71-PB [5]. As a positive binding control, we used a soluble form of recombinant P-selectin (P-selectin-Fc). P-selectin-Fc did not bind to any PSGL-1 transfectants in the presence of 2 mM EDTA (Fig. 1B). P-selectin-Fc bound weakly to 293T cells transiently expressing PSGL-1 (293T/PSGL-1) in the presence of Ca^2+^ but not to 293T/T57A cells (Fig. 1B). Double expression of PSGL-1 and FUT7 in 293T cells resulted in the efficient binding of P-selectin-Fc to PSGL-1 in a calcium-dependent manner (Fig. 1B). Even in the presence of Ca^2+^ and FUT7, P-selectin-Fc did not bind to 293T/T57A cells (Fig. 1B). These observations are consistent with previous findings that interaction of PSGL-1 with P-selectin is calcium-dependent and requires appropriate *O*-glycosylation of PSGL-1 at T57 [10], [12]. In contrast, EV71-1095 showed marked binding to 293T/PSGL-1 cells in a calcium-independent manner, even in the absence of FUT7 (Fig. 1B). EV71-1095 also bound to 293T/T57A cells (Fig. 1B). These results indicate that, unlike the interaction between PSGL-1 and P-selectin, the interaction between PSGL-1 and EV71-1095 does not require Ca^2+^ and the *O*-glycans at T57 of PSGL-1.

### Sialic acids are not necessary for EV71-1095 binding

**Figure 2:**
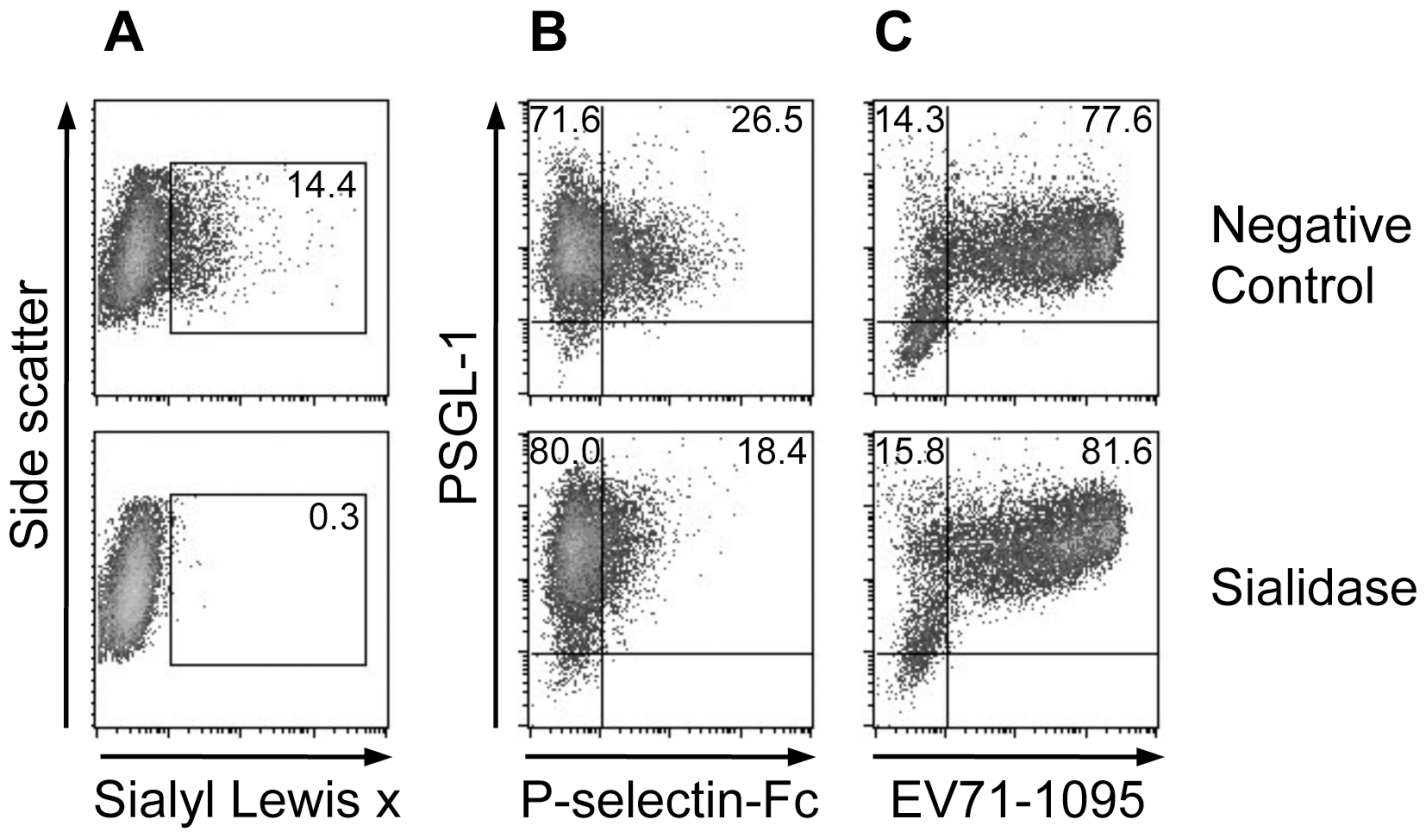
Effect of sialidase treatment on PSGL-1 binding to EV71-1095. (A) Sialyl Lewis x expression on the cell surface, as measured with flow cytometry. The percentage of cells expressing sialyl Lewis x is indicated. (B) The cells were examined with the P-selectin-Fc binding assay using flow cytometry. The percentage of cells bound to P-selectin-Fc is indicated in the upper right quadrant. (C) The cells were examined with the EV71 binding assay using flow cytometry. The percentage of cells bound to EV71-1095 is indicated in the upper right quadrant. As a negative control, 293T/PSGL-1 cells were incubated in the medium without sialidase. The data are representative of three independent experiments.

To examine the role of sialic acids on the cell surface, including sialyl Lewis x moieties in the potential *O*-glycans at T44 and T57 of PSGL-1, on EV71 binding to 293T/PSGL-1 cells, we tested EV71 binding to the cells pretreated with sialidase. Sialidase treatment removed cell-surface sialyl Lewis x (Fig. 2A) and reduced P-selectin-Fc binding to 293T/PSGL-1 cells (Fig. 2B). On the other hand, EV71-1095 binding to the sialidase-treated cells was not reduced regardless of the removal of sialyl Lewis x (Fig. 2C). Although treatment with sialidase decreased EV71 infection to DLD-1 cells [21], sialic acids on the cell surface of 293T/PSGL-1 cells are not necessary for the binding of PSGL-1 to EV71-1095.

### An inhibitor of sulfation reduces PSGL-1 binding to EV71-PB

**Figure 3:**
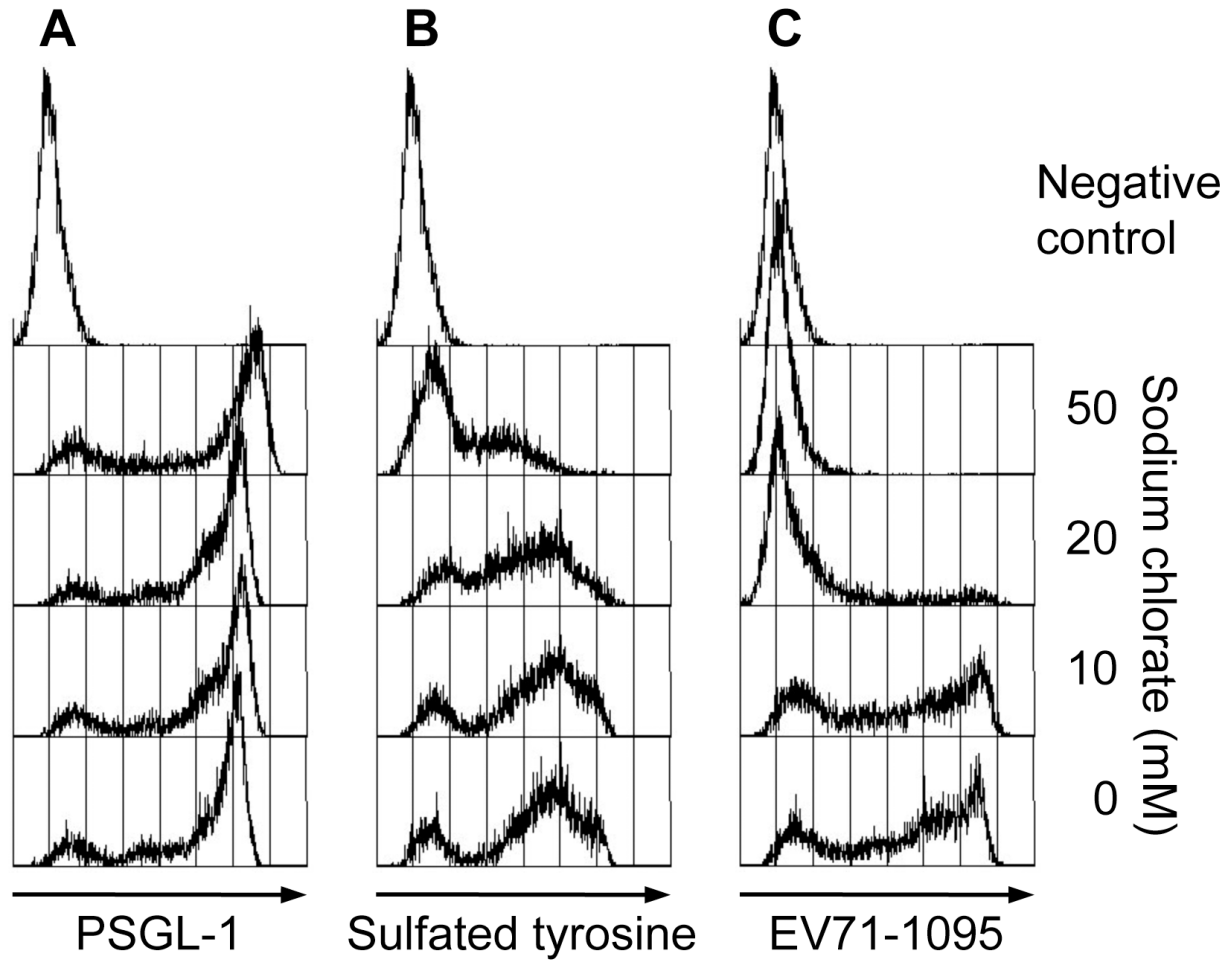
Effect of sodium chlorate on PSGL-1 binding to EV71-1095. Pretreatment of 293T/PSGL-1 cells with sodium chlorate reduces EV71-1095 binding in a dose-dependent manner. (A) PSGL-1 expression on the cell surface, as measured with flow cytometry. As a negative control, 293T/PSGL-1 cells cultured in the absence of sodium chlorate were stained with an isotype control antibody. (B) Sulfated tyrosines on the cell surface, as measured with flow cytometry. As a negative control, 293T/PSGL-1 cells cultured in the absence of sodium chlorate were stained with an isotype control antibody. (C) The cells were examined with the EV71 binding assay using flow cytometry. As a binding control, 293T/PSGL-1 cells were treated with mock-infected culture supernatant. The data are representative of three independent experiments.

In addition to *O*-glycosylation of PSGL-1, sulfation of the three tyrosines (Y46, Y48, and Y51) in the N-terminal region of PSGL-1 is required for high affinity binding to P- and L-selectins [13], [14], [15], [22], [23]. To assess the role of tyrosine sulfation of PSGL-1 in the PSGL-1–EV71 interaction, we treated 293T/PSGL-1 cells with sodium chlorate, an inhibitor of sulfation that blocks PSGL-1 binding to P-selectin [13]. As described previously, sodium chlorate had no apparent effect on PSGL-1 expression on the cell surface (Fig. 3A). On the other hand, sodium chlorate reduced sulfated tyrosines on the cell surface (Fig. 3B) and inhibited EV71-1095 binding to 293T/PSGL-1 cells in a dose-dependent manner (Fig. 3C). These observations indicated that sulfation of PSGL-1, in addition to its expression on the cell surface, is important for EV71 binding.

### One or more tyrosines in the N-terminal region of PSGL-1 are important for EV71-PB binding

**Figure 4:**
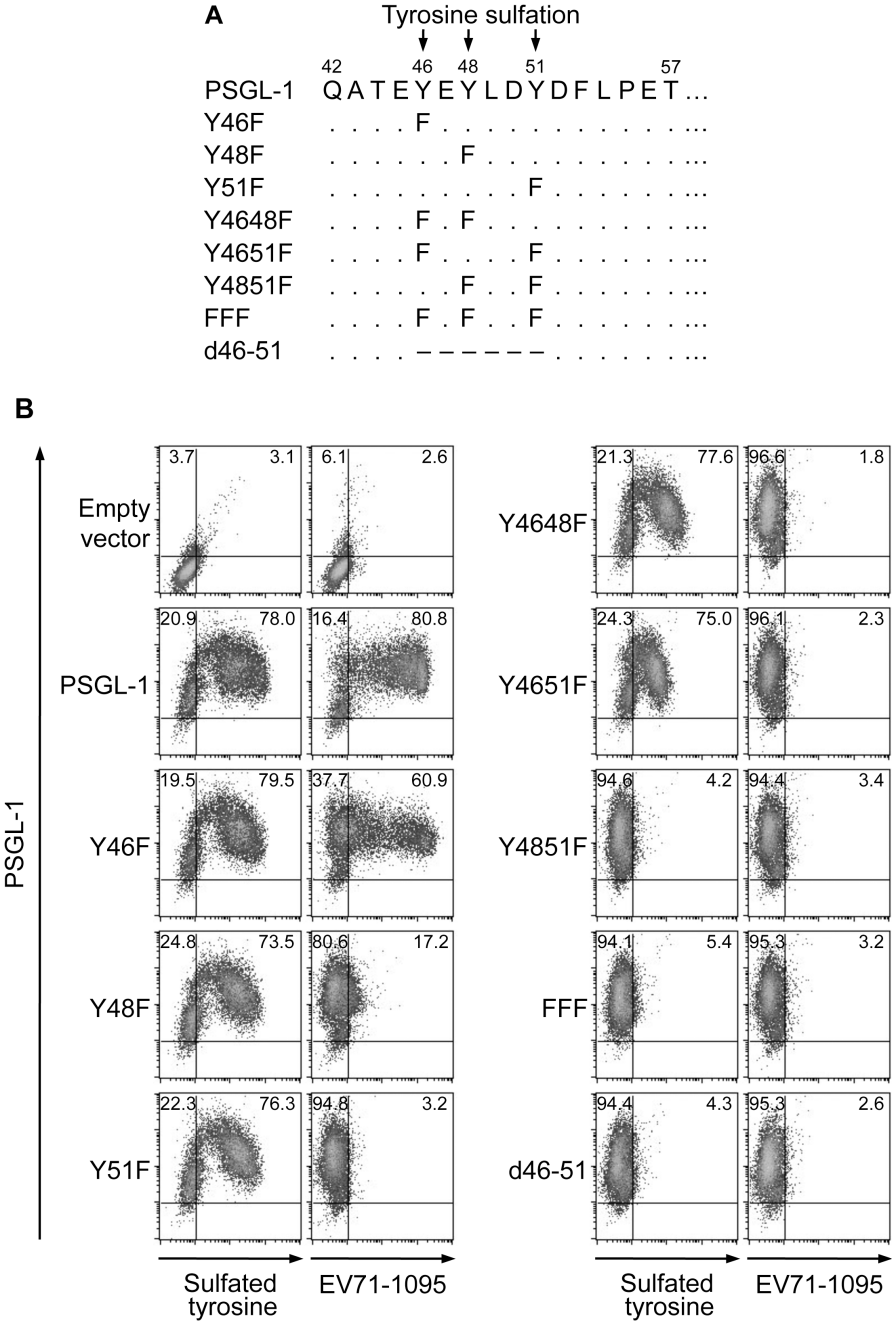
Sulfated tyrosines in the N-terminal region of PSGL-1 are important for binding to EV71-1095. (A) Putative sites of tyrosine sulfation (Y46, Y48, and Y51) in the N-terminus (aa 42–57) of PSGL-1 and the series of PSGL-1 mutants constructed. Identical and deleted amino acids are indicated by dots (.) and dashes (–), respectively. (B) EV71-1095 binding to PSGL-1 mutants. The percentage of cells expressing tyrosine sulfated proteins or bound to EV71-1095 is indicated in the upper right quadrant. The data are representative of three independent experiments.

We then determined the requirement for the putative sulfated tyrosines (Y46, Y48, or Y51) in the N-terminal region of PSGL-1 for its binding to EV71-1095. We generated PSGL-1 mutants with phenylalanine substitutions at one or more tyrosines and a mutant with a deletion of this region (Fig. 4A). We transfected 293T cells with expression plasmids containing the PSGL-1 mutants and used them for the EV71 binding assay using flow cytometry. 293T cells transfected with an empty vector expressed little or no detectable tyrosine sulfated proteins on the cell surface (Fig. 4B). Similar to the binding of PSGL-1 to P-selectin [13], [14], substitution of the tyrosines with phenylalanine prevented tyrosine sulfation and PSGL-1 binding to EV71-1095 (Fig. 4B). Substitution of one or two tyrosines slightly reduced (Y46F) or impaired (Y48F, Y51F, Y4648F, or Y4651F) the binding of PSGL-1 to EV71-1095 regardless of the apparent expression of tyrosine sulfated proteins on the cell surface (Fig. 4B). Substitution of two or three tyrosines (Y4851F or FFF) or deletion of the region (d46–51) reduced tyrosine sulfated proteins on the cell surface and completely disrupted the PSGL-1–EV71 interaction (Fig. 4B). We also examined the role of tyrosine sulfation in PSGL-1 binding to other EV71-PB strains. Binding of SK-EV006, C7/Osaka, KED005, and 75-Yamagata strains to 293T/PSGL-1 cells was also inhibited by sodium chlorate (Fig. S1). These strains bound to 293T/T57A cells but not to 293T cells expressing the PSGL-1-FFF mutant. Taken together, these findings demonstrate that, in contrast to *O*-glycosylation at T57, tyrosine sulfation of PSGL-1 is essential for the efficient binding to EV71-PB strains.

### Sodium chlorate inhibits EV71-PB replication in Jurkat T cells

**Figure 5:**
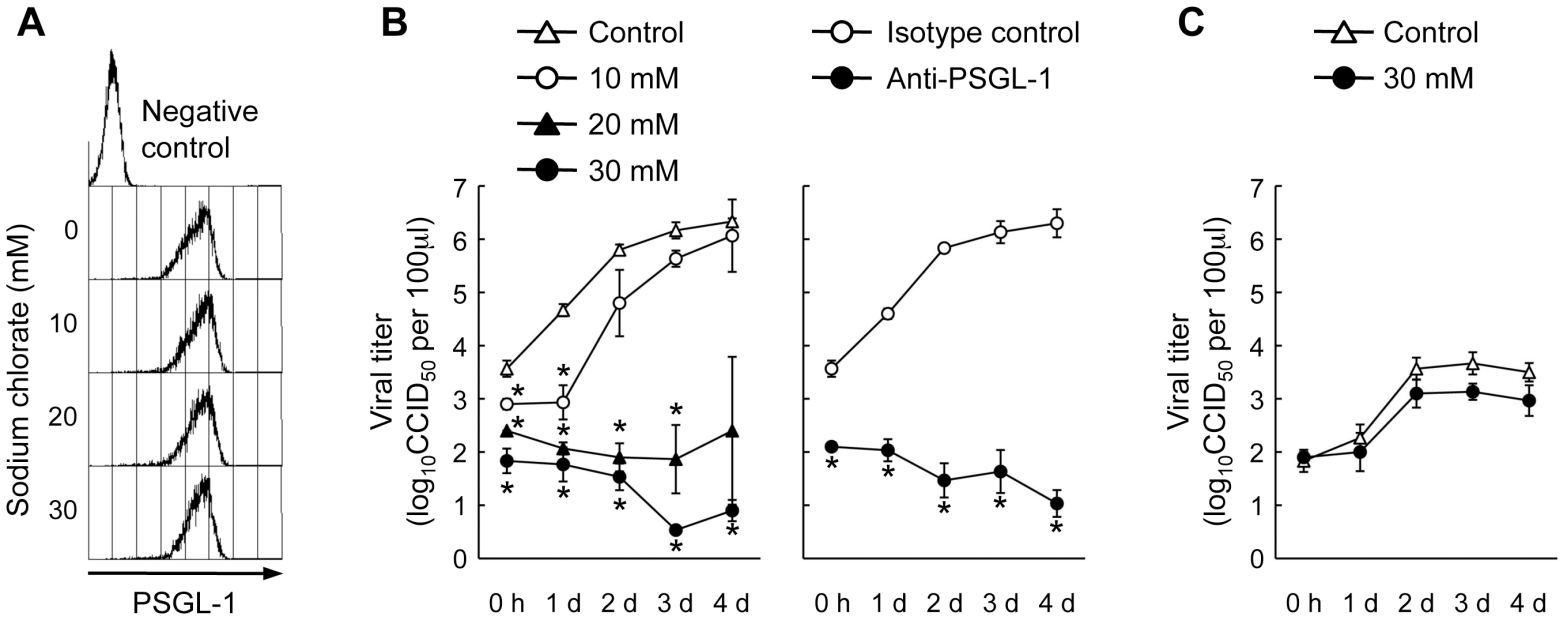
Effect of sodium chlorate on EV71-1095 replication in Jurkat T cells. (A) PSGL-1 expression on the Jurkat T cell surface, as measured by flow cytometry. As a negative control, Jurkat T cells maintained in the absence of sodium chlorate were stained with an isotype control antibody. (B) EV71-1095 growth kinetics in Jurkat T cells in the presence of sodium chlorate. Viral titers were determined at 0 h, 1 day, 2 days, 3 days, and 4 days after EV71-1095 inoculation in Jurkat T cells. As a control for inhibition of EV71 replication, EV71-1095 growth kinetics in Jurkat T cells in the presence of anti-PSGL-1 (KPL1) and control antibodies are shown. (C) EV71-02363 (EV71-non-PB) growth kinetics in Jurkat T cells in the presence of sodium chlorate. Viral titers are indicated as the mean ± S.D. of triplicate analyses. Asterisks indicate *P*<0.01 compared to those of the controls.

**Figure 6:**
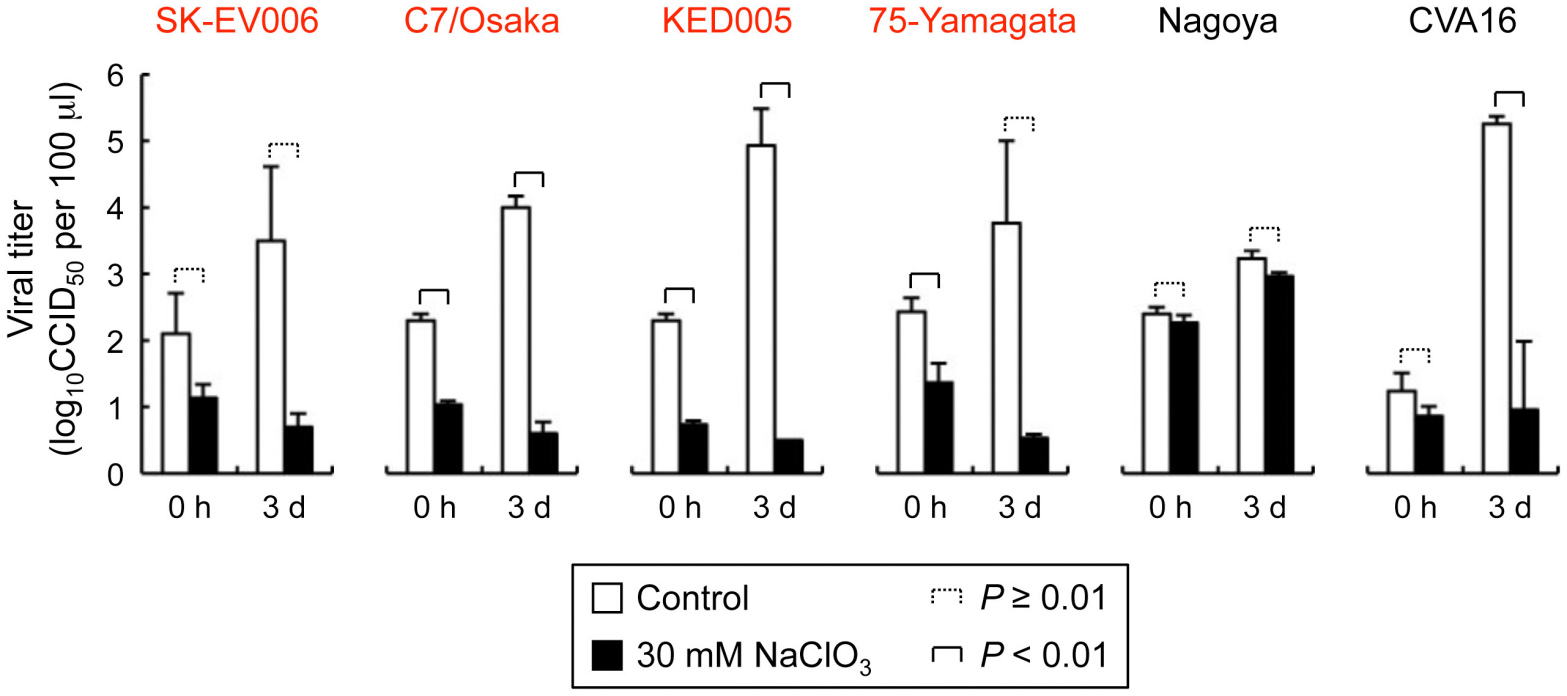
Replication of five EV71 strains and coxsackievirus A16 (CVA16) in Jurkat T cells in the presence of sodium chlorate. Viral replication was determined in Jurkat T cells incubated with 30 mM sodium chlorate. EV71-PB strains are indicated in red. Titers are expressed as the mean, and error bars indicate the S.D. of triplicate or quintuplicate (CVA16) analyses.

We next examined whether sulfation of PSGL-1 is required for PSGL-1-dependent replication of EV71-PB in Jurkat T cells. Jurkat T cells were infected with EV71 and cultured in the presence of sodium chlorate to inhibit the sulfation of PSGL-1. Sodium chlorate treatment did not affect PSGL-1 expression on Jurkat T cells (Fig. 5A). On the other hand, sodium chlorate significantly inhibited the replication of EV71-1095 in a dose-dependent manner (Fig. 5B). The replication of other EV71-PB strains was also inhibited in the presence of sodium chlorate (Fig. 6). In contrast, replication of EV71-non-PB strains (EV71-02362 and EV71-Nagoya), which can replicate in Jurkat T cells in a PSGL-1-independent manner [5], was not affected by sodium chlorate (Figs. 5C and 6). This observation supports that sodium chlorate inhibited replication by blocking EV71-PB entry into the cells. To confirm that sodium chlorate is acting at the receptor level, we transfected Jurkat T cells with genomic RNA of EV71-1095 and examined viral titers at 24 h posttransfection in the presence or absence of 30 mM sodium chlorate. Although infectious viruses were recovered in the presence of sodium chlorate, the mean viral titer in the presence of sodium chlorate was over 10 times lower than that of the control experiments (data not shown). Although sodium chlorate inhibited EV71-PB-binding to PSGL-1 expressing cells (Figs. 3C and 5B (0 h postinfection)), we could not rule out the possible involvement of the sodium chlorate treatment during the later stages of viral replication. Further studies are needed to elucidate the inhibitory mechanism of action of sodium chlorate in a receptor dependent or independent manner during different stages of viral replication of EV71.

Replication of the G-10 strain of coxsackievirus A16, which may use another unidentified receptor(s) to infect Jurkat T cells [5], [24], was significantly inhibited by sodium chlorate (Fig. 6). This result suggests that some sulfated molecules other than PSGL-1 might be involved in the replication of coxsackievirus A16 in Jurkat T cells in a PSGL-1-independent manner.

## Discussion

We have shown that tyrosine sulfation, but not *O*-glycosylation, of the N-terminal region of PSGL-1 is critical for EV71-PB binding to PSGL-1 and for virus entry and subsequent replication of EV71-PB in Jurkat T cells. First, unlike P-selectin-Fc, EV71-PB bound to a PSGL-1 mutant with an alanine substitution at the potential *O*-glycosylation site (T57) in a calcium-independent manner (Figs. 1 and S1). Second, removal of sialyl Lewis x by sialidase did not reduce PSGL-1 binding to EV71 (Fig. 2). Third, a sulfation inhibitor, sodium chlorate, significantly impaired EV71-PB binding to PSGL-1 in a dose-dependent manner (Figs. 3 and S1). Fourth, EV71-PB binding to PSGL-1 was inhibited when phenylalanine substitutions were made at one or more potential tyrosine sulfation sites in the N-terminal region of PSGL-1 (Figs. 4 and S1). Finally, PSGL-1-dependent viral replication of EV71-PB strains in Jurkat T cells, but not EV71-non-PB strains, was inhibited by sodium chlorate (Figs. 5 and 6).

Human PSGL-1 is one of the most characterized tyrosine sulfated proteins at the molecular level [11]. The involvement of *O*-glycans and sulfated tyrosines in the structural and functional basis of PSGL-1 binding to its natural ligands has been extensively studied, and distinct requirements for tyrosine sulfation for PSGL-1 binding to selectins have been elucidated. Among the three potential sulfated tyrosines of human PSGL-1, Y46 and Y51, but not Y48, are important for PSGL-1 binding to L-selectin along with a core-2 based *O*-glycan with sialyl Lewis x at T57 [22]. On the other hand, the crystal structure of the lectin and EGF domains of P-selectin co-complexed with the N-terminal domain of PSGL-1 revealed a critical involvement of sulfated tyrosines at Y48 and Y51 for direct molecular contact with P-selectin [11]. The corresponding interactions via sulfated tyrosines are not formed in E-selectin binding in the crystal structure of the PSGL-1–E-selectin complex [11]. Thus, tyrosine sulfation is critical for PSGL-1 binding to L- and P-selectins, but not to E-selectin [14]. In our study, we have shown that sulfated tyrosines at Y48 and Y51 play a critical role in PSGL-1 binding to EV71-PB. However, *O*-glycosylation at T57 and sialyl Lewis x moieties on the potential *O*-glycans of PSGL-1 were not required for the PSGL-1–EV71 interaction, suggesting distinct structural requirements between EV71 and P-selectin for PSGL-1 binding. To elucidate the structural basis of the PSGL-1–EV71 interaction, further studies will be needed to identify genetic determinants in EV71 capsid proteins required for PSGL-1 binding using both EV71-PB and non-PB strains.

Yang et al. [21] have recently reported that EV71 may use sialylated glycans as receptors for infection in intestinal DLD-1 cells. In our current study, we showed that potential *O*-glycans at T57 and sialic acids are not critical for binding to EV71-PB (Figs. 1 and 2). However, our study does not exclude possible contributions of sialic acids and other proteins with or without *O*-glycans on the cell surface of various cells during the course of EV71 replication in a PSGL-1-dependent or -independent manner [21], [24], [25].

In contrast to the structural requirements of *O*-glycans for PSGL-1 binding to selectins, all three sulfated tyrosines, but not *O*-glycans at T57, are required for PSGL-1 binding with the skin-associated chemokine, CCL27 [9]. PSGL-1 facilitates P-selectin-mediated T cell migration in the inflamed skin [26], [27] and interacts with the chemokine CCL27 to regulate skin-homing T cells [9]. HFMD pathogenesis due to EV71 can be characterized as acute skin inflammation. Therefore, it is possible that binding of EV71-PB with PSGL-1-positive skin-homing T cells and/or Langerhans cells, and subsequent viral replication in those cells, may participate in HFMD pathogenesis and progression. The status of tyrosine sulfation of PSGL-1 on those cells may modulate cell migration and PSGL-1-dependent replication of EV71-PB in the inflamed skin.

An important role for tyrosine sulfation of a specific cellular receptor in viral entry and replication has been demonstrated for the first time in a co-receptor for HIV-1, CCR5 [18]. CCR5 is a functional receptor for macrophage inflammatory protein (MIP)-1α and MIP-1β, and is expressed on memory/effector T cells, macrophages, and immature dendritic cells [28]. The N-terminal region of CCR5 is highly modified by tyrosine sulfation and *O*-glycosylation, and sulfated tyrosines play critical roles in CCR5 interactions with chemokines [18]. Site-directed mutagenesis and treatment with sodium chlorate revealed that sulfation of tyrosine residues in the N-terminal region of CCR5 is required for efficient CCR5 binding to MIP-1α and MIP-1β, and to HIV-1 gp120-CD4 complexes, without affecting the expression of CCR5 [18]. Likewise, the efficacy of HIV-1 entry was significantly reduced in cells expressing CCR5 mutants with one or more phenylalanine substitutions at four potential tyrosine sulfated residues compared to that in cells expressing native CCR5 [18]. Tyrosine sulfation may be a common phenomenon in chemokine receptors expressed on immune cells such as leukocytes, platelets, and dendritic cells [16]. Therefore, tyrosine sulfation seems to regulate not only the migration of immune cells but also the infectivity of viruses.

Although the occurrence of severe EV71 infection with a number of fatal cases mainly in children continues to be a major public health threat in the Asia Pacific region, no vaccines or antiviral agents are currently available for EV71 [29]. Our data suggest that the virus-receptor interaction may be a promising target for potential antiviral agents. Thus, soluble PSGL-1 as one such agent may have an inhibitory effect on EV71-PB replication [5]. In our current study, we have demonstrated the possible involvement of tyrosine sulfation of PSGL-1 on EV71 entry into target cells, and accordingly, we showed the inhibitory effect of a tyrosine sulfation inhibitor on viral replication of EV71-PB strains in Jurkat T cells. Thus, the elucidation of the structural and functional basis of virus-receptor interactions will provide novel and unique antiviral approaches for the treatment of severe EV71-associated diseases.

## Materials and Methods

### Cells

293T cells were maintained in Dulbecco's modified Eagle's medium (DMEM, Wako) supplemented with 10% fetal calf serum (FCS). Jurkat T cells were maintained in RPMI medium (Sigma) supplemented with 10% FCS.

### Viruses

All EV71 strains (Table 1) and the coxsackievirus A16 prototype strain (G-10) were propagated in RD or Vero cells. Because some of the strains produced diffuse plaques on RD cells, the viral titers were determined by a microtitration assay using 96-well plates and RD cells, as previously described [30]. Briefly, 10 wells were used for each viral dilution, and the viral titers were expressed as 50% cell culture infectious dose (CCID_50_). For flow cytometry, we used concentrated viruses unless otherwise stated. To prepare virus concentrations, viruses were ultracentrifuged, and the amount of EV71 virions was measured.

**Table 1:** EV71 strains.

Strain (Subgenogroup)	PSGL-1 binding phenotype1)	Accession No.	Reference
SK-EV006 (B3)	PB	AB059819	[33]
C7/Osaka (B4)	PB	AB059818	[33]
KED0052) (C1)	PB		[33]
1095 (C2)	PB	AB059817	[30], [34]
75-Yamagata (C4)	PB	AB177813	[35]
Nagoya (B1)	Non-PB	AB059813	[36]
02363 (C1)	Non-PB	AB115495	[34]

1) PB: PSGL-1–binding, Non-PB: PSGL-1–non-binding [5].

2) The VP1 nucleotide sequence of KED005 is identical to that of the 03784-MAA-97 strain (accession no. AY207612) isolated in Malaysia [37].

### Antibodies and recombinant proteins

The anti-EV71 monoclonal antibody (mAb) MA105 (mouse IgG_2b_) was generated from mice immunized with EV71-1095 (Y. Tano et al., unpublished data) Immunization to mice, fusion, selection of hybridomas, and propagation of hybridomas in the ascite fluid of the mice, were outsourced to Nippon Biotest Laboratories Inc., Tokyo, Japan. The anti-human PSGL-1 mAb KPL1 and anti-sialyl Lewis x mAb CSLEX1 were purchased from BD Biosciences. Anti-human PSGL-1 mAb PL2 was purchased from Beckman–Coulter. Anti-sulfotyrosine mAb Sulfo-1C-A2 [31] was purchased from Millipore. For the negative control, mouse IgG_1_ (MOPC-21) and IgG_2a_ (G155–178) were purchased from BioLegend and BD Biosciences, respectively. Recombinant P-selectin-Fc was purchased from R&D Systems.

### Plasmids and mutagenesis

For directional cloning using a *Cpo*I recognition site [32], we introduced a *Cpo*I recognition-compatible (*San*DI) site into the pcDNA3.1(+) plasmid (Invitrogen). The *Bam*HI-*Eco*RI fragment of pcDNA3.1(+) was replaced with 5′-ggatccgggtcccggtaagaattc-3′ (*Bam*HI+*San*DI+gg+Stop+*Eco*RI) to produce pcDNA3.1SS. Human *FUT7* cDNA was amplified from Jurkat T cell cDNA with the primers FUT7-F1 (5′-atacggtccggccatgaataatgctgggcacggc-3′) and FUT7-R1 (5′-tgacggaccgtcaggcctgaaaccaaccct-3′). The *FUT7* ORF was sub-cloned into a *San*DI site in pcDNA3.1SS to produce pcDNA-FUT7. The sequence of the cloned *FUT7* ORF was identical to that of *FUT7* (NM_004479).

The primers used for mutagenesis/deletion are provided in Table S1. Briefly, cDNA of human *SELPLG* was cloned into pEF6-Flag-3S [5] to produce pEF-PSGL-1 [5]. Mutations and deletions were introduced into the N-terminal region of human PSGL-1 with PCR, and the mutated *SELPLG* cDNA was cloned into pEF6-Flag-3S.

### Transfection of 293T cells

293T cells were transfected with expression plasmids using Lipofectamine 2000 (Invitrogen), and DMEM medium was replaced with fresh medium 4 h after transfection. The cells were collected 24 h after transfection by pipetting, and were used for flow cytometry. For inhibition of tyrosine sulfation of PSGL-1, 293T cells were treated with 10–50 mM sodium chlorate in DMEM for 1 day. Four hours after transfection with pEF-PSGL-1, the medium was replaced with medium containing sodium chlorate, and the cells were further incubated for 20 h.

### Flow cytometry

The cells were washed once with flow cytometry buffer (FC buffer; PBS(−) supplemented with 2 mM EDTA, 2% FCS, and 0.1% NaN_3_) and incubated with the indicated mAb on ice for 30 min. After washing with FC buffer, the cells were incubated with secondary antibodies conjugated with Alexa Fluor 488 (Invitrogen). To detect sialyl Lewis x, the cells were incubated with secondary antibodies conjugated with R-phycoerythrin (SouthernBiotech). To detect PSGL-1 by two-color flow cytometry, PL2 was labeled with a Zenon mouse IgG_1_ R-phycoerythrin labeling kit (Invitrogen). To detect P-selectin-Fc binding, PBS(−) supplemented with 2 mM CaCl_2_, 2% FCS, and 0.1% NaN_3_ was used instead of FC buffer. The cells were washed and analyzed with FACSCalibur (Becton Dickenson).

### EV71-binding assay by flow cytometry

293T cells (5×10^5^) transfected with the indicated expression plasmid were washed once with FC buffer and incubated with the EV71-1095 preparation (1×10^7^ CCID_50_) supplemented with 0.1% NaN_3_, or concentrated viruses (containing 0.5 µg of VP1 protein) per 50 µl FC buffer. The cells were washed and stained for 30 min on ice with Alexa Fluor 488-conjugated MA105.

### Sialidase treatment of cells

Cells were processed as in the EV71-binding assay and flow cytometry described above. Prior to the addition of EV71, P-selectin-Fc, or mAb, cells (2.5×10^6^) were incubated with 50 mU/ml of *Vibrio cholerae* sialidase (Roche) in 500 µl of DMEM supplemented with 2% FCS for 1 h at 37°C and then washed once.

### Viral infection assays

Jurkat T cells (4×10^4^ cells) were inoculated with viruses at 1 CCID_50_/cell for 1 h on ice, washed, and incubated in medium (200 µl in a 48-well plate) at 34°C. For inhibition of tyrosine sulfation of PSGL-1, the cells were pretreated with 10–30 mM sodium chlorate in medium for more than 3 days, inoculated with viruses, washed, and maintained in medium supplemented with sodium chlorate. For mAb inhibition, the cells were pretreated with 10 µg/ml mAb for 1 h, washed, and maintained in medium with 10 µg/ml mAb. At the indicated time, the infected cells and supernatants were freeze-thawed, and viral titers were determined by CCID_50_ titration in RD cells. All infection assays were carried out in triplicate unless otherwise stated, and the mean viral titers were compared using Student's *t*-test (two-tailed). *P* values<0.01 were considered statistically significant.

## Supporting Information

Table S1 Substitution/deletion mutant primers.
(0.04 MB DOC)

Figure S1 Binding of four EV71-PB strains to 293T cells expressing PSGL-1. 293T cells were transfected with the indicated expression plasmids (wild-type PSGL-1, T57A, or FFF) and cultured in the absence (PSGL-1, T57A, and FFF) or presence (PSGL-1+NaClO_3_) of 50 mM sodium chlorate. The transfectants were incubated with concentrated EV71 and used for the EV71 binding assay using flow cytometry. As a negative control, cells were incubated with concentrated supernatant from the RD cell culture (RD sup.). The percentage of cells bound to EV71 is indicated in the upper right quadrant.
(2.14 MB TIF)
