# Supplementary material for: Tyrosine Sulfation of the Amino Terminus of PSGL-1 Is Critical for Enterovirus 71 Infection
Source: PLoS Pathog. 2010 Nov 4;6(11):e1001174. doi: 10.1371/journal.ppat.1001174 (PMC2973824; doi:10.1371/journal.ppat.1001174)
Supplement: Table S1 — Substitution/deletion mutant primers. (0.04 MB DOC) [file ppat.1001174.s001.doc]

**Table S1. Substitution/deletion mutant primers.**

| Construct | S/A^1)^ | Sequence (5'—3')^2)^ |
| --- | --- | --- |
| PSGL-1/T57A | S | ctgccagaaGcggagcctccagaaatgct |
|  | A | ttctggaggctccgCttctggcaggaaatc |
| PSGL-1/Y46F | S | tTtgagtacctagattatgatttcctgccagaa |
|  | A | ataatctaggtactcaAattcggtggcctgtctcc |
| PSGL-1/Y48F | S | tatgagtTcctagattatgatttcctgccagaa |
|  | A | ataatctaggAactcatattcggtggcctgtctcc |
| PSGL-1/Y51F | S | tatgagtacctagattTtgatttcctgccagaa |
|  | A | aAaatctaggtactcatattcggtggcctgtctcc |
| PSGL-1/Y4648F | S | tTtgagtTcctagattatgatttcctgccagaa |
|  | A | ataatctaggAactcaAattcggtggcctgtctcc |
| PSGL-1/Y4651F | S | tTtgagtacctagattTtgatttcctgccagaa |
|  | A | aAaatctaggtactcaAattcggtggcctgtctcc |
| PSGL-1/Y4851F | S | tatgagtTcctagattTtgatttcctgccagaa |
|  | A | aAaatctaggAactcatattcggtggcctgtctcc |
| PSGL-1/FFF | S | tTtgagtTcctagattTtgatttcctgccagaaac |
|  | A | aAaatctaggAactcAaattcggtggcctgtctcc |
| PSGL-1/d46-51 | S | ggccaccgaagatttcctgccagaaacgga |
|  | A | gcaggaaatcttcggtggcctgtctccggt |

^1)^S, sense; A, antisense.

^2)^Substituted nucleotides are indicated by uppercase letters.
