# Supplementary figures and images for: Tyrosine Sulfation of the Amino Terminus of PSGL-1 Is Critical for Enterovirus 71 Infection
Source: PLoS Pathog. 2010 Nov 4;6(11):e1001174. doi: 10.1371/journal.ppat.1001174 (PMC2973824; doi:10.1371/journal.ppat.1001174)

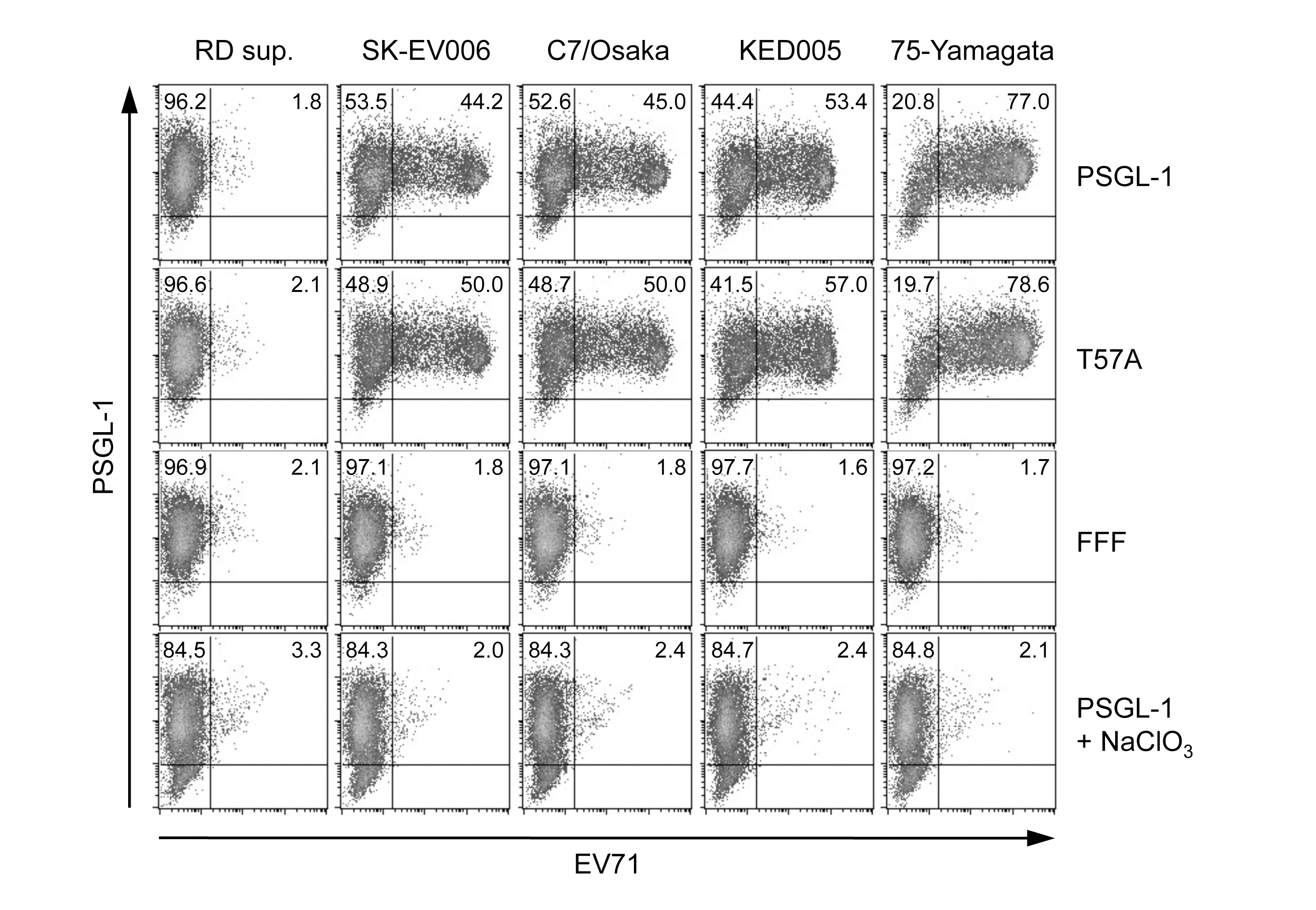

Supplement: Figure S1 — Binding of four EV71-PB strains to 293T cells expressing PSGL-1. 293T cells were transfected with the indicated expression plasmids (wild-type PSGL-1, T57A, or FFF) and cultured in the absence (PSGL-1, T57A, and FFF) or presence (PSGL-1+NaClO3) of 50 mM sodium chlorate. The transfectants were incubated with concentrated EV71 and used for the EV71 binding assay using flow cytometry. As a negative control, cells were incubated with concentrated supernatant from the RD cell culture (RD sup.). The percentage of cells bound to EV71 is indicated in the upper right quadrant. (2.14 MB TIF) [file ppat.1001174.s002.tif]
